# Identification of Male-Specific Markers by Genotyping-by-Sequencing in the Giant Spiny Frog, *Quasipaa spinosa*

**DOI:** 10.3390/genes16111347

**Published:** 2025-11-07

**Authors:** Yu Xiao, Yun Xia, Xiaomao Zeng

**Affiliations:** 1 Chengdu Institute of Biology, Chinese Academy of Sciences, Chengdu 610213, China; xiaoyu@cib.ac.cn; 2 University of Chinese Academy of Sciences, Beijing 100049, China

**Keywords:** GBS, sex-specific markers, sex determination, sex differentiation

## Abstract

Background/Objectives: *Quasipaa spinosa*, a large-sized spiny frog, has high commercial value in the food trade. Although the sexual dimorphism in body size between males and females has been investigated, the sex-determining mechanism in this frog remains unknown. Methods: This study employed a genotyping-by-sequencing (GBS) method to identify sex-associated genomic markers and elucidate the sex determination mechanism in the species. Results: We obtained 853 candidate sex-specific GBS tags, with 811 tags (95.07%) demonstrating a male heterozygous system (XX/XY). The diagnostic specificity of the nine markers was further demonstrated by PCR analysis across multiple adult individuals from seven distinct geographic populations of the frog. Four sex-specific markers were aligned with the *DMRT1* gene, representing a master regulator of sex determination and gonadal differentiation across the animal kingdom. Conclusions: Our results deciphered the genetic mechanisms governing sex determination in *Q. spinosa* and presented effective strategies for mono-sex breeding.

## 1. Introduction

In contrast with mammals and birds, the sex determination mechanism of most amphibians is remarkably diverse, with homomorphic and/or little-differentiated sex chromosomes [1,2,3]. Frogs exhibit diverse sex-determination mechanisms, with documented systems including the male heterogametic XX/XY model and the female heterogametic ZZ/ZW model, along with variants, such as X1X2X1X2/X1X2Y and 00/0W [4,5].

Although only one master sex-determining gene, the *DMW* (*DMRT1* paralog) in the African clawed frog *Xenopus laevis*, was known within amphibians [6], the *BOD1L* gene in the green toad *Bufo viridis* was newly discovered as a Y-specific candidate sex determination locus [7]. *DMRT1* has been reported as a putative male-determining gene in the common frog *Rana temporaria* and the European tree-frog *Hyla arborea* group [8,9]. In addition, there are several potential candidates, being sex-specific, related to sex determination/gonadal differentiation in frogs, such as genes implicated in feminization with *CYP19*, *SF1*, *FOXL2*, and *SOX3*, and in masculinization with *AMH*, *AR*, and *CYP17* [10,11,12,13,14]. All the above suggests that sex-determining genes display lineage-specific divergence across frog species.

*Quasipaa spinosa*, one of the large-sized spiny frogs, belonging to the family Dicroglossidae, mainly live in low-elevation mountain streams of South China [15]. This species has high economic value for its delicious meat and potential medicinal features [16], triggering the expansion in artificial cultivation, with an annual output now exceeding 6000 tons in China, showing significant market potential [17].

The giant spiny frog needs one to two years to reach first maturation, and the male frog matures a year earlier than the female [18]. Its mature male size exhibits advantages over females [19,20,21]. The sexual dimorphism, particularly male-biased growth rates, supports the implementation of breeding an all-male population as a cost-effective strategy to maximize production efficiency and operational sustainability. Subsequently, the development of a mono-sex culture fundamentally requires understanding the sex determination mechanism. According to the sex determination system, sex control breeding could be realized by sex manipulation biotechnology via distinguishing gender [22].

The identification of sex-specific markers not only facilitates characterizing genetic sex determination (GSD) systems and distinguishing genetic sexes, but also providing critical clues for pinpointing master regulatory genes through genome-wide association studies. To overcome challenges posed by the large genome size of amphibians, genotyping-by-sequencing (GBS) has been widely adopted for efficient identification of multiple sex-specific markers. To date, sex-specific markers have been explored from more than 20 ranid frogs, and used for identification of sex determination systems and/or sex chromosomes through GBS/RAD-seq [23,24,25,26,27,28]. Female-specific markers were successfully produced by GBS/RAD-seq in the Chinese giant salamander *Andrias davidianus* [29]. Sex-specific markers enable efficient genetic sex identification, particularly during early developmental stages. In the giant spiny frog, morphological differentiation of sexes remains challenging prior to sexual maturation due to the absence of secondary sexual characteristics. The establishment of juvenile-stage genetic sex identifiers is critical for implementing precision breeding strategies, enabling early sex diagnosis to optimize monosex aquaculture and sex reversal protocols in this species.

The sex determination system of the giant spiny frog (*Q. spinosa*) remains poorly understood. To address this knowledge gap, we employed GBS to identify sex-associated single nucleotide polymorphisms (SNPs), subsequently validating these sex-specific markers through PCR assays across geographically distinct populations in China. The sex determination/differentiation genes were explored through mapping sex-specific markers onto annotated gene sequences. Our investigations revealed the mechanism of sex determination and established foundational molecular resources for developing genetic sex identification protocols in the giant spiny frog, and also provided a basis for mono-sex breeding in the future.

## 2. Materials and Methods

### 2.1. Sampling and Preparation

A total of 67 adult *Q. spinosa* specimens were collected across seven natural populations in China, with geographic distribution details and population codes documented in Supplementary File S1. Histological examination of gonadal tissues was employed to confirm physiological sex. Muscle tissue samples from both sexes were preserved in 95% ethanol at −20 °C for downstream genomic analyses. Among these, 20 specimens from YH were selected for GBS-sequencing, comprising 10 males and 10 females.

All specimen collection procedures were conducted under permit CIB-2017009, approved by the Animal Care and Use Committee of the Chengdu Institute of Biology (Chinese Academy of Sciences), in compliance with ethical guidelines for amphibian research.

### 2.2. DNA Extraction and Genotyping-by-Sequencing

Genomic DNA was isolated from skeletal muscle tissue using the Qiagen^®^ DNeasy Blood and Tissue Kit (QIAGEN, Valencia, CA, USA) following the manufacturer’s animal tissue protocol. Approximately 500 mg of muscle tissue was first flash-frozen in liquid nitrogen and mechanically homogenized to a fine powder. Afterward, the powder was digested enzymatically with proteinase K at 56 °C for one hour. Subsequent purification was performed on 20 mg aliquots of the homogenized tissue, yielding genomic DNA that was finally eluted in 100 μL of nuclease-free aqueous buffer.

The GBS libraries were prepared following Elshire et al. [30] with modifications: (1) restriction digestion of genomic DNA using *MseI* endonuclease (New England Biolabs, Ipswich, MA, USA) at 37 °C, followed by barcode adapter ligation; (2) PCR amplification of digested fragments; (3) size-selected (300–350 bp) through agarose gel electrophoresis; (4) gel-purified using Gel Extraction Kit (QIAGEN, Valencia, CA, USA).

Libraries were sequenced on an Illumina NovaSeq 6000 platform (San Diego, CA, USA) generating 150 bp paired-end reads. To obtain high-quality clean reads, the raw sequencing data were subjected to quality control processing, which included the removal of adapters and barcodes.

### 2.3. Filtering and SNP Calling

Upon acquisition of raw data from GBS, quality control was conducted using the fastp software (V0.22.0). The filtering criteria included the exclusion of reads containing more than 10% undetermined bases (N) and reads where low-quality bases (Q ≤ 5) constituted over 50% of the sequence. The data needed to meet a Q30 threshold of greater than 85%. After that, we employed Stacks v2.41 for the analysis of GBS data [31]. In the initial stage of data processing, we utilized the *process_radtags* module to conduct demultiplexing and quality filtering. This module effectively removed low-quality reads and those without restriction sites, ensuring the integrity and reliability of the subsequent analysis.

The subsequent genomic analysis was conducted using the de novo assembly pipeline in Stacks, with three critical parameters implemented: minimum stack depth (m = 2), intra-individual mismatches (M = 2), and inter-individual mismatches (n = 1).

### 2.4. Sex-Specific Locus Discovery and Isolation

We implemented three bioinformatic approaches adapted from Brelsford et al. [23] to identify sex-specific markers, which are based on three characteristic features of sex-specific markers located on heterogametic sex chromosomes, with XY systems serving as an example and the female heterozygous subsystem (ZW) as the opposite.

The initial method involved the identification of SNP loci by analyzing allele frequency disparities between sexes. In XY systems, females are expected to possess two copies of sex-specific SNPs, whereas males have only one. Due to potential sequencing artifacts and recombination events, we designated SNPs as sex-specific markers under the following criteria: loci showing near-fixation (≥0.95) in females and inter-sex allele frequency divergence ≥ 0.4. The calculation of allele frequency quantification across sexes was conducted via the Stacks-2.41 population module.

The secondary screening approach detected sex-specific SNPs by examining sex-specific heterozygosity patterns. Candidate loci were considered sex-specific based on specific criteria derived from the *populations.sumstats.tsv* output. Specifically, a locus was classified as sex-specific when it demonstrated complete homozygosity in at least 50% of female individuals (F_hom ≥ 0.5) and heterozygosity in at least 50% of male individuals (M_het ≥ 0.5).

The third approach focused on sex-limited locus detection that was exclusive to a particular sex. From the output of the Stacks software (V2.41), sexually dimorphic presence/absence patterns were analyzed for each GBS loci under this approach, and a locus meeting both conditions was classified as male-specific markers when it was entirely absent in females and detected in at least 50% of male individuals.

### 2.5. Confirmation of Sex Chromosome and Sex-Specific Markers

We used the direct mapping method to determine the chromosomal location of sex-specific markers. The sex-specific markers were directly mapped to the reference genome by using BLAST (V2.12.0) to determine relative positions of the sex-specific markers on the *Q. spinosa* genome [32,33]. The top matches with e-values no more than 1 × 10^−10^ were retained.

### 2.6. PCR Validation

To validate the authenticity of the identified sex-specific markers and distinguish them from potential individual-specific artifacts in the GBS data, we implemented PCR amplification coupled with gel electrophoretic separation across all available samples. This experimental validation approach, while demonstrated here for an XX/XY system, is equally applicable for investigating ZZ/ZW sex determination systems. Our experimental design incorporated two distinct primer strategies.

**Figure 1:**
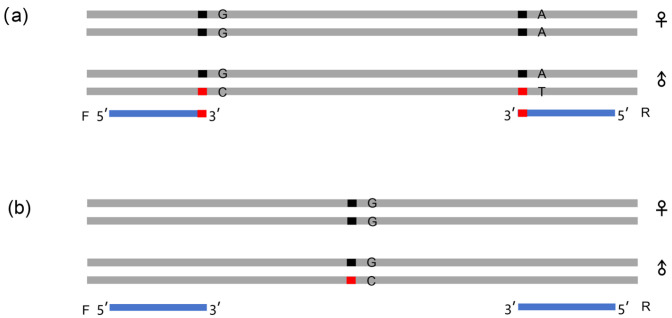
Two types of primer design for sex-specific markers. Examples suggest an XX/XY sex chromosome system, but the results are similar in species with a ZZ/ZW system. The blue line indicates primers, and ‘F’ and ‘R’ indicate the forward primer and reverse primer, respectively. Red segments indicate Y-specific SNPs (i.e., C and T) that do not occur on the X. (**a**) PA: Both the forward and reverse primers were designed at Y-specific SNPs. The Y-specific base was set as the first base of the 3′ of the primer sequence. (**b**) SD: Both the forward and reverse primers were designed at the male-specific sequence.

For male-specific presence/absence (PA) markers, we developed an allele-specific primer design strategy targeting male-restricted loci. Both upstream and downstream primers were designed with diagnostic SNPs positioned at the 3′ terminal nucleotide to prevent cross-amplification of female homologs (Figure 1a). Electrophoretic separation of the target sequences was carried out using a 1.5% agarose gel. Following gel electrophoresis, we anticipated observing a band in males, while no band would be present in females.

The other primer was employed to amplify a sexually dimorphic SNP locus, referred to as a single-nucleotide differential sex-specific marker (SD). This differentiation was confirmed through sequencing. We established a co-dominant marker system through conserved flanking primer design in sex-specific regions (Figure 1b). PCR products spanning polymorphic sites were subjected to capillary sequencing at the Sangon Sequencing Center (Shanghai, China). Sequence alignment analysis revealed sexually dimorphic nucleotide states, with heterozygous genotypes in males contrasting homozygous patterns in females at target loci.

### 2.7. Predicting the Genes for Sex Determination

We initiated the process by BLAST to map the coding regions of conserved candidate sex-determining genes onto the *Q. spinosa* genome, thereby anchoring their chromosomal localization (E-value ≤ 1 × 10^−10^). Subsequently, we enhanced the alignment regions through extension and trimming. Finally, we aligned these refined regions with sex-specific loci to evaluate whether any sex-specific loci were present within them. Building upon previous studies that identified potential sex-determining genes (including *AMH*, *AMHR2*, *AR*, *CYP17*, *CYP19A1*, *RSPO1*, *BOD1L*, *DMRT1*, *FOXL2*, *SOX3*, and *SF1*), we employed a comparative genomics approach to evaluate whether our identified sex-specific markers correspond to these candidate genes.

## 3. Results

### 3.1. GBS Data Analysis and SNP Calling

Genotyping-by-sequencing (GBS) of 20 adult specimens (10 males, 10 females) generated 242.76 million paired-end raw Illumina sequencing reads. After utilizing the process_radtags tool to eliminate low-quality sequences, 241,851,458 reads were retained, with an average number of 12,344,970 in females and 11,840,175 in males. Sequencing coverage across loci ranged from 8.64× to 13.59×, ensuring high reliability for robust identification of candidate sex-specific markers (Supplementary Table S1). The Stacks *de novo*_*map* pipeline was then employed for variant calling, yielding a catalog of 8,144,505 genome-wide SNPs.

### 3.2. Discovery and Isolation of Sex-Specific Loci

Using frequency differences, we detected 545 GBS-tags (containing 713 sex-specific SNPs) that displayed genotype distributions consistent with a male heterogametic (XX/XY) system. Notably, no loci matched a female heterogametic (ZZ/ZW) pattern, strongly suggesting absence of ZW-linked markers in the studied species. Conversely, the heterozygosity-based method identified 545 XY-patterned GBS-tags with 1426 SNPs located on it, in which 713 SNPs were also found by the first approach. Like the first method, this approach did not uncover any ZW-linked SNPs. Using the sex-limited occurrence method, 266 male-specific and 42 female-specific GBS tags were identified. In total, the three approaches revealed 853 putative sex-specific GBS-tags, with 95.07% (811/853, binomial test, *p* ≤ 0.01) of these GBS-tags strongly suggesting an XX/XY sex-determination system in *Q. spinosa* (Supplementary Table S2).

### 3.3. Confirmation of Sex-Specific Markers

The candidate sex-specific markers derived from the STACKS genotyping was performed through comparative alignment against the *Q. spinosa* genome. Among the 811 putative XY-linked GBS tags identified, 685 (84.5%) were successfully mapped onto chromosome 1 and confirmed as sex-specific markers (Supplementary Table S3).

### 3.4. Development and Validation of Sex-Specific Markers

Nine randomly selected sex-specific markers were PCR-verified, with detailed primer information provided in Table 1.

**Figure 2:**
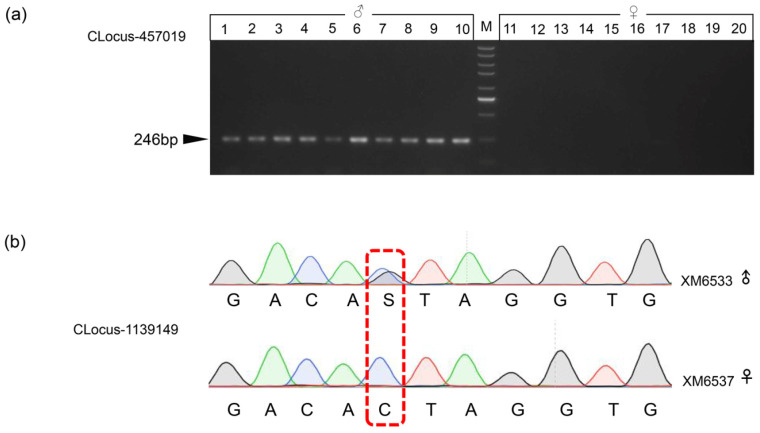
Validation of sex-specific markers. (**a**) Gel electrophoresis showing the PCR amplification of sex-specific marker CLocus-457019. The locus ID is indicated on the left. ‘M’ indicates a DNA marker. Black arrows indicate the PCR product size. (**b**) The sequencing peaks of sex-specific marker CLocos-1139149 in male (XM6533) and female (XM6537) individuals after PCR amplification. The male shows double peaks at SNP sites (S = G/C), while the female exhibits a single peak.

Three sex-specific markers were used to design PA primers, and PCR amplification of these loci in 20 adult samples (10 males and 10 females) from the YH population revealed 100% concordance between gel banding patterns. We successfully amplified male-specific bands in all ten males, while no amplification was observed in the ten females (see details in Figure 2a and Figure S1). However, these three sex-specific markers could not be validated in other populations.

The remaining six sex-specific markers were specifically employed in constructing single nucleotide differential (SD) primers targeting sexually dimorphic SNP loci. Following the amplification of all samples from seven populations, the male individuals exhibited heterozygosity, while the female individuals displayed homozygosity at their polymorphic sites (Figure 2b). All six sex-specific markers were confirmed across seven populations, displaying varied geographic distributions. Some markers exhibited sex linkage in specific population sets, while others revealed overlapping associations within the same populations. CLocus_1139149 exhibited universal validation across all populations, whereas CLocus_862681 was restricted to YH and DH populations. The other three sex-specific markers (CLoci 328282, 4136690, 4142019) showed consistent validation in TS, YZ, and YH populations (see details in Figure S2 and Supplementary Table S4).

This pan-population validation study confirms the diagnostic reliability of the nine sex-specific markers, which exhibited consistent amplification fidelity across all 67 specimens representing seven geographically distinct populations.

### 3.5. Identifying Potential Sex-Determining Genes

Using indirect methods, only the *Glandirana ruguosa DMRT1* gene is mapped uniquely to chromosome 1. The alignment revealed discordant genomic mapping patterns for the remaining candidate sex-determination genes in *Q. spinosa*, with either failed alignment (E-value > 1 × 10^−20^) or ambiguous chromosomal localization across multiple linkage groups, thus precluding their classification as sex-determination loci. BLAST analysis identified four sex-specific markers (CLoci 2728, 48294, 50898, 1454664) within the *DMRT1* alignment region of the *Q. spinosa* genome (Figure S3, Supplementary Tables S5 and S6). Based on the alignment results, the *DMRT1* gene may be a potential candidate sex-determining gene in *Q. spinosa*.

## 4. Discussion

Our data show robust evidence that the giant spiny frog *Q. spinosa* has a male-heterogametic sex determination system. It is supported by our findings that 811 out of 853 GBS tags analyzed (95.07%) exhibited male-specific patterns (Supplementary Table S2). Nine randomly selected sex-specific loci (Table 1) originally identified through genome-wide differentiation scans were validated by a PCR test with 67 additional individuals from seven different geographic sites of this frog (Supplementary Table S3). Collectively, these markers provided conclusive validation of an XX/XY sex determination system through multilocus genotyping analyses in *Q. spinosa*.

These nine sex-specific markers revealed significant spatial heterogeneity in marker efficacy across seven geographic populations. Specifically, one pan-regional marker demonstrated 100% site applicability (7/7), followed by markers with decreasing geographic fidelity (6/7, 5/7, 4/7, 4/7, 2/7), while three markers exhibited strict site-specificity (1/7). This cline in cross-population transferability highlights the necessity of hierarchical marker selection protocols for broad-scale conservation applications (Supplementary Table S4). Among these sex-specific markers, five loci can distinguish the genetic sex of individuals across most sites spanning the primary distribution range of the species *Q. spinosa*. Their cross-population reliability suggests strong evolutionary conservation, positioning them as robust candidates for implementing sex control protocols in conservation breeding programs and aquaculture management strategies. Here, a polymorphism in sex-specific markers was found in different geographic populations of this frog. This intraspecific variation is consistent with the patterns observed from other frog species of *Rana temporaria*, *R. clamitans*, *R. dybowskii*, *H. arborea*, *Quasipaa boulengeri*, and *Amolops* species [25,34,35,36]. It is because most amphibians have little-differentiated sex chromosomes, thus displaying in parallel with incomplete recombination suppression between X and Y chromosomes across different geographic populations [37].

Sex-specific markers are useful for screening genes involved in sex determination and differentiation. When these sex-specific markers can be mapped to the genes on the sex-limited chromosomes, it becomes possible to identify and isolate the genes that are actively involved in the processes of sex determination and differentiation. Our data show that four male-specific markers were matched with the transcription factor *DMRT1* of the frog *G. ruguosa* [38], and this gene was located on the sex chromosome pair of the *Q. spinosa* (Figure S3; Supplementary Tables S5 and S6). *DMRT1* is essential for male differentiation and evolutionarily conserved across metazoans. *DMRT1*, along with its orthologs/paralogs, serves as a key gene in sex determination across a wide range of taxa, including birds, the fruit fly *Drosophila melanogaster* (*dsx*), the nematode *Caenorhabditis elegans* (*mab-3*), the zebrafish *Danio rerio* (*zfDmrt1*), the medaka fish *Oryzias latipes* (*DMY*), and the clawed frog *X. laevis* (*DMW*) [6,39,40,41,42,43]. In various anuran lineages, such as Hylid frogs, and the ranid frogs *R. temporaria* and *Rana japonica*, *DMRT1* has been identified as sex-specific or recognized as a significant gene in sexual differentiation [9,14,44]. The sex-specific markers associated with the *DMRT1* gene in the present study reveal its potential as a candidate sex determination gene in the giant spiny frog *Q. spinosa*. This finding further validates the observation that the *DMRT1* gene family plays a fundamental and widespread role in sex determination and gonadal differentiation processes across species. Exploring whether *DMRT1* functions as the putative master sex determination gene in *Q. spinosa* represents an exciting and potential focus for future investigations.

Our data exhibited sex-specific SNPs matched to Chromosome 1. This was backed by the high mapping rate, with 685 out of 811 sequences (84.5%) aligning successfully with the *Q. spinosa* genome. Chromosome 1, the largest chromosome containing the gene *DMRT1*, has been independently recruited for sex determination in several anuran lineages, such as species of hylid frogs and true frogs [24,44]. Now, it has been extended to spiny frogs, newly found in family Dicroglossidae. Interestingly, chromosome 3 was also assigned to be the sex chromosome in the Shimen population from Hunan province of the *Q. spinosa*, with the sexual genotype conducted using whole-genome re-sequencing [45]. Definitively, this is different from our results by using the genotyping-by-sequencing (GBS) method. Thus, the sex-determining locus was shown on non-homologous chromosomes among different populations of the same species. Actually, this situation was also found in other anuran species, such as *G. ruguosa*, *R. japonica*, *Rana sakuraii*, and *Rana tagoi* [24,46,47]. For this sex chromosome transition, it often comes in a group with homomorphic sex chromosomes. This phenomenon might be explained by the recruitment of a new gene from a non-sex chromosome, which translocated to and replaced the existing sex-determining gene [48,49].

The sex-specific markers with PCR-based sex identification provide an effective tool for mono-sexual population control in commercial aquaculture operations. In frogs, the phenotypic sex can be changed by the exogenous hormones during the early development stage [50,51,52]. Then, with exogenous hormone treatment, we can generate the phenotypic male (XX) or female (XY). The sex-specific markers can distinguish the genetic sex, thus giving the possibility to choose these phenotypic females (XY) to mate with normal males (XY). Subsequently, we could obtain offspring individuals with a genetic female (XX), male (XY), and super-male (YY). Finally, we can establish an all-XY population via using a super-male (YY) to mate with a female (XX). Utilizing sex-specific marker screening, sex control breeding has been successfully implemented for some commercial fishes [53,54,55]. In frogs, the window period of hormone treatment would be in the larval stage of tadpoles for inducing sex reversal individuals (i.e., XX male or XY female) [50,52]. Obviously, using morphological traits to determine the sex is challenging during this tadpole stage. Instead, sex-specific markers, developed in the present study, make it possible to separate the sex of larvae for the giant spiny frog. For the PCR amplification of these markers, just a swabbing sample of skin is required for extracting genome DNA without damage [56,57,58].

## 5. Conclusions

The male heterogametic system (XX/XY) in the giant spiny frog *Q. spinosa* was successfully validated, by using the GBS approach via screening sex-specific markers. Nine male-specific markers were successfully developed and verified by the PCR test in a number of adult individuals from seven geographic populations of this frog. The transcription factor *DMRT1* was matched with sex-specific markers investigated here, considered to be a putative candidate sex determination/differentiation gene. Our findings provide evidence suggesting an XX/XY sex determination system in *Q. spinosa*, and give the potential for advancing sex control technologies in the giant spiny frog.

## Figures and Tables

**Table 1:** Primer sequences of nine sex-specific markers isolated from *Q. spinosa*.

Locus	Primer Sequence (5′–3′)	TM (°C)	Length (bp)	Site Type
457019	F: CAGACAACAAAATCCGTATTGCA	58	246	PA
	R: TTTCCTTGGTAATCCCGTCTT			
316336	F: CAGGATGGCCATCAGATTTATACTC	58	251	PA
	R: TTACCTTCTTCTTTAGATAGTGTG			
696744	F: TTGTGGTGCATTGAAACACATGA	60	107	PA
	R: GTGTTCAAGACCCCACAATCTACTA			
1508254	F: TAATGAAATGTGTTTGGTCTAA	55	275	SD
	R: ATGTCCCCACACAGTCAAAT			
328282	F: CTGCTGTGGGTCTCCGAT	55	217	SD
	R: CTGTCTTCGCCGCGTGCT			
4136690	F: AGGCAGGAGCAATGTGTCA	56	229	SD
	R: TGAATCTAAGCCACAATAATG			
4140219	F: TTTCCCAAAGTGGTGCCTAT	56	241	SD
	R: TATATTCTCTTTAGTTCAGGCC			
1139149	F: TAAGAATGCTGTAAATGGTAGACTA	58	267	SD
	R: TAGTGCAACTTTGTTTCATTCAT			
862681	F: GGGTACAATAAAGTCAAGTAGGCT	58	233	SD
	R: GAGTGTCTGCATATGTTGGTGT			

## Data Availability

Raw sequence reads generated in this study are archived under accession number CRA024998 in the China National Centre for Bioinformation (CNCB) database (https://ngdc.cncb.ac.cn/gsa/, accessed on 22 October 2025).

## Supplementary Materials

The following supporting information can be downloaded at https://www.mdpi.com/article/10.3390/genes16111347/s1, File S1: Sample size and location information. Figure S1: Gel electrophoresis showing the PCR amplification of two PA sex-linked markers; Figure S2: The sequencing results of six SD sex-linked markers in seven populations; Figure S3: The mapping of the sex-linked locus on the *DMRT1* gene of *G. rugosa*; Table S1: Quality summary of *Q. spinosa* by GBS; Table S2: Detailed information of all putatively sex-linked markers screened from *Q. spinosa*; Table S3: The results of mapping sex-linked markers onto the genome of *Q. spinosa*; Table S4: The results of validation of six SD sex-linked markers in seven populations; Table S5: Blast results of candidate sex-determining genes to the reference genome; Table S6: Mapping the sex-linked loci to the alignment region of *DMRT1* in the genome of *Q. spinosa*.
