# Supplementary material for: Identification of Male-Specific Markers by Genotyping-by-Sequencing in the Giant Spiny Frog, Quasipaa spinosa
Source: Genes (Basel). 2025 Nov 7;16(11):1347. doi: 10.3390/genes16111347 (PMC12652585; doi:10.3390/genes16111347)
Supplement: Supplementary file 1 [file genes-16-01347-s001.zip › Figure S1..pdf]

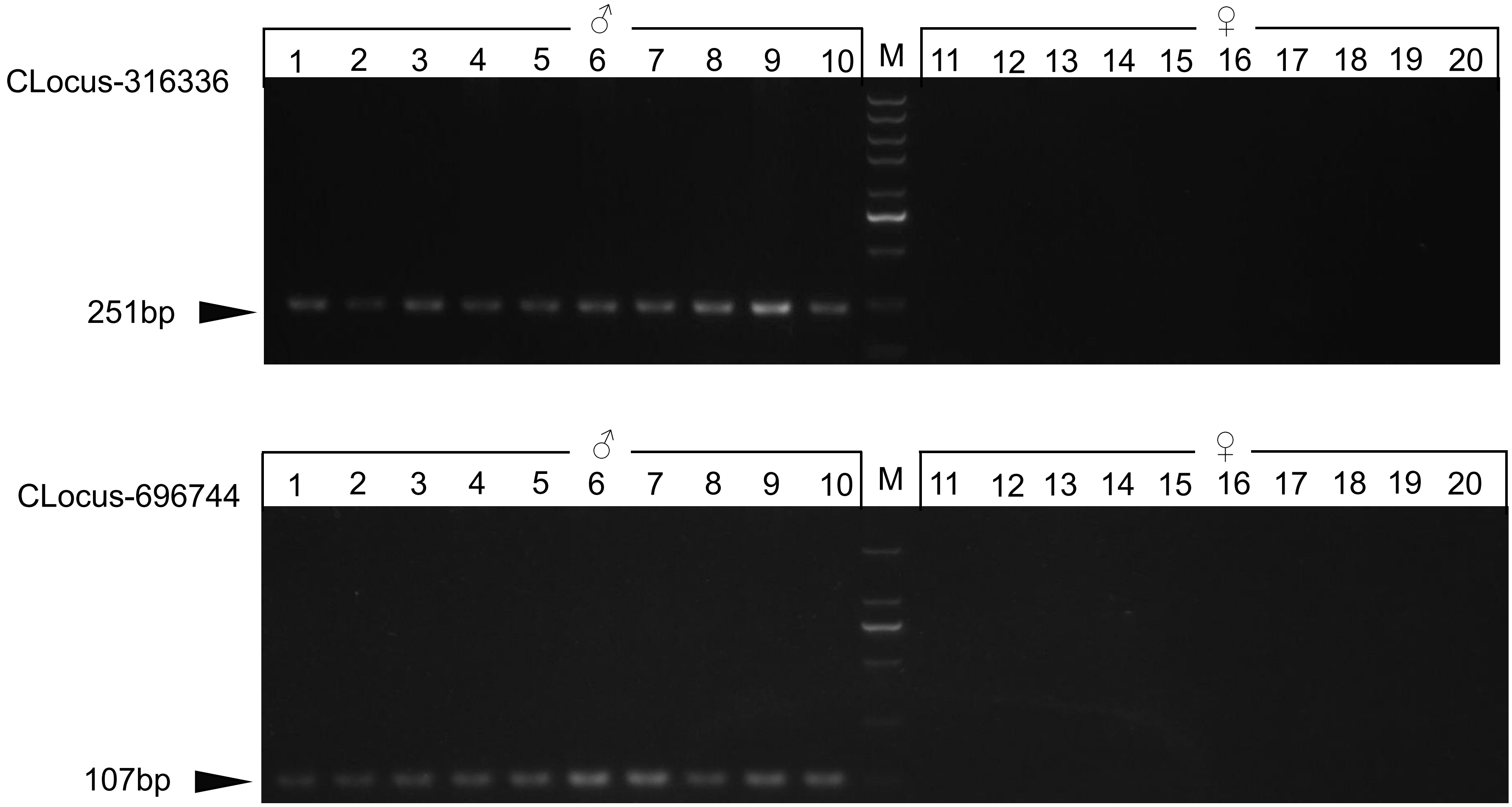

Figure S1. Gel electrophoresis showing the PCR amplification of two PA sex-linked markers (CLocus-316336, CLocus-696744).The locus ID is indicated to the left. ‘M’ indicates a DNA marker. Black arrows indicate the PCR products size.
