# Supplementary material for: Identification of Male-Specific Markers by Genotyping-by-Sequencing in the Giant Spiny Frog, Quasipaa spinosa
Source: Genes (Basel). 2025 Nov 7;16(11):1347. doi: 10.3390/genes16111347 (PMC12652585; doi:10.3390/genes16111347)
Supplement: Supplementary file 1 [file genes-16-01347-s001.zip › Figure S2..pdf]

Figure S2. The squencing results of six SD sex-linked markers in seven populations. Each code consists of population and sample information. For example, in "TS-XM6562_M", "TS" is the population code representing Tongshan County, "XM6562" is the sample number, and "M" indicates a male individual. More populations code details could be seen in file S1. R, S, W, and Y are degenerate bases. R stands for A/G. S stands for G/C. W stands for A/T. Y stands for C/T.

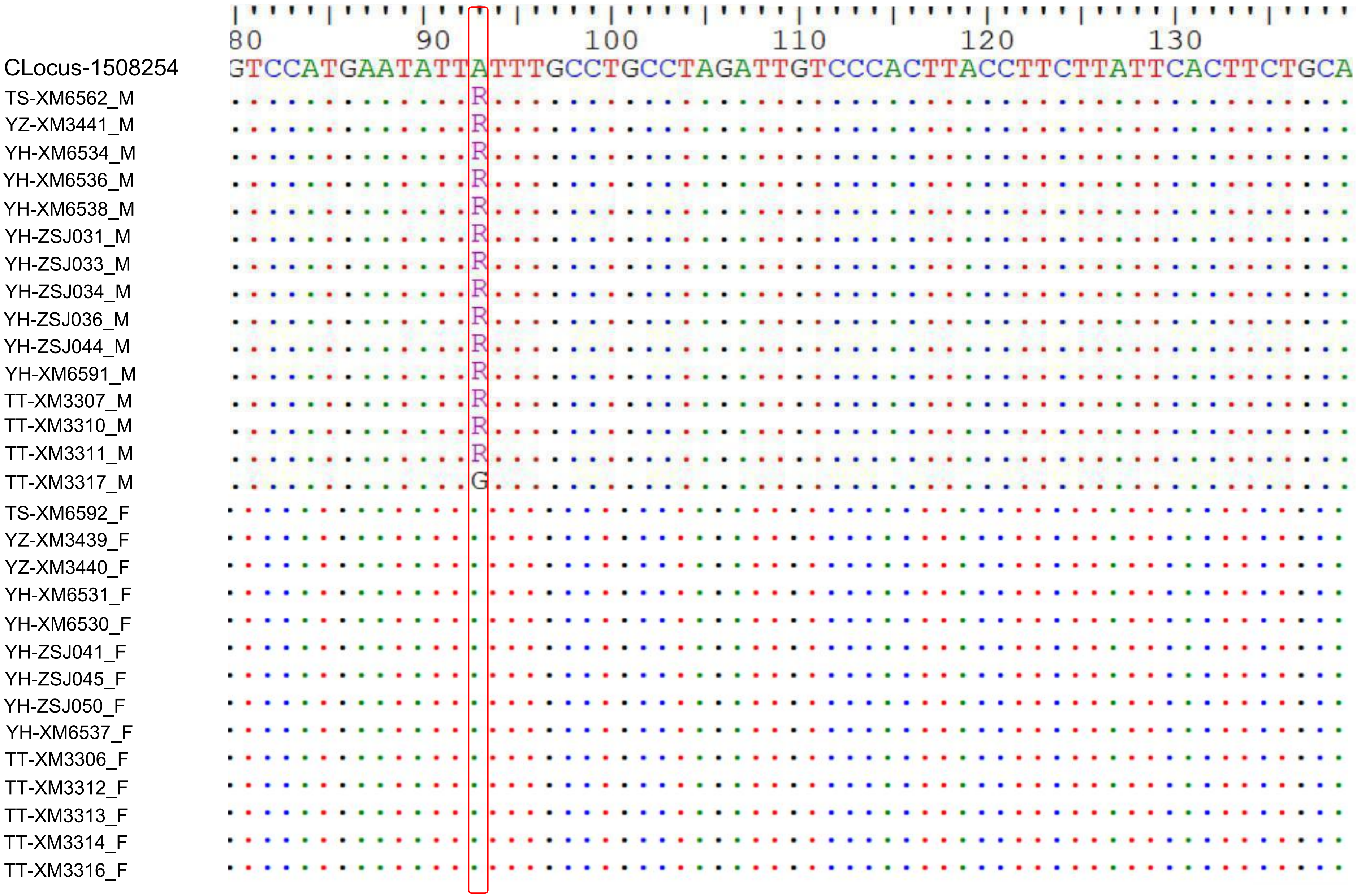
80
90
100
110
120
130
CLocus-1508254
GTCCATGAATATTATTTGCCTGCCTAGATTGTCCCACTTACCTTCTTATTCACTTCTGCA
TS-XM6562_M
YZ-XM3441_M
YH-XM6534_M
YH-XM6536_M
YH-XM6538_M
YH-ZSJ031_M
YH-ZSJ033_M
YH-ZSJ034_M
YH-ZSJ036_M
YH-ZSJ044_M
YH-XM6591_M
TT-XM3307_M
TT-XM3310_M
TT-XM3311_M
TT-XM3317_M
TS-XM6592_F
YZ-XM3439_F
YZ-XM3440_F
YH-XM6531_F
YH-XM6530_F
YH-ZSJ041_F
YH-ZSJ045_F
YH-ZSJ050_F
YH-XM6537_F
TT-XM3306_F
TT-XM3312_F
TT-XM3313_F
TT-XM3314_F
TT-XM3316_F
R
G

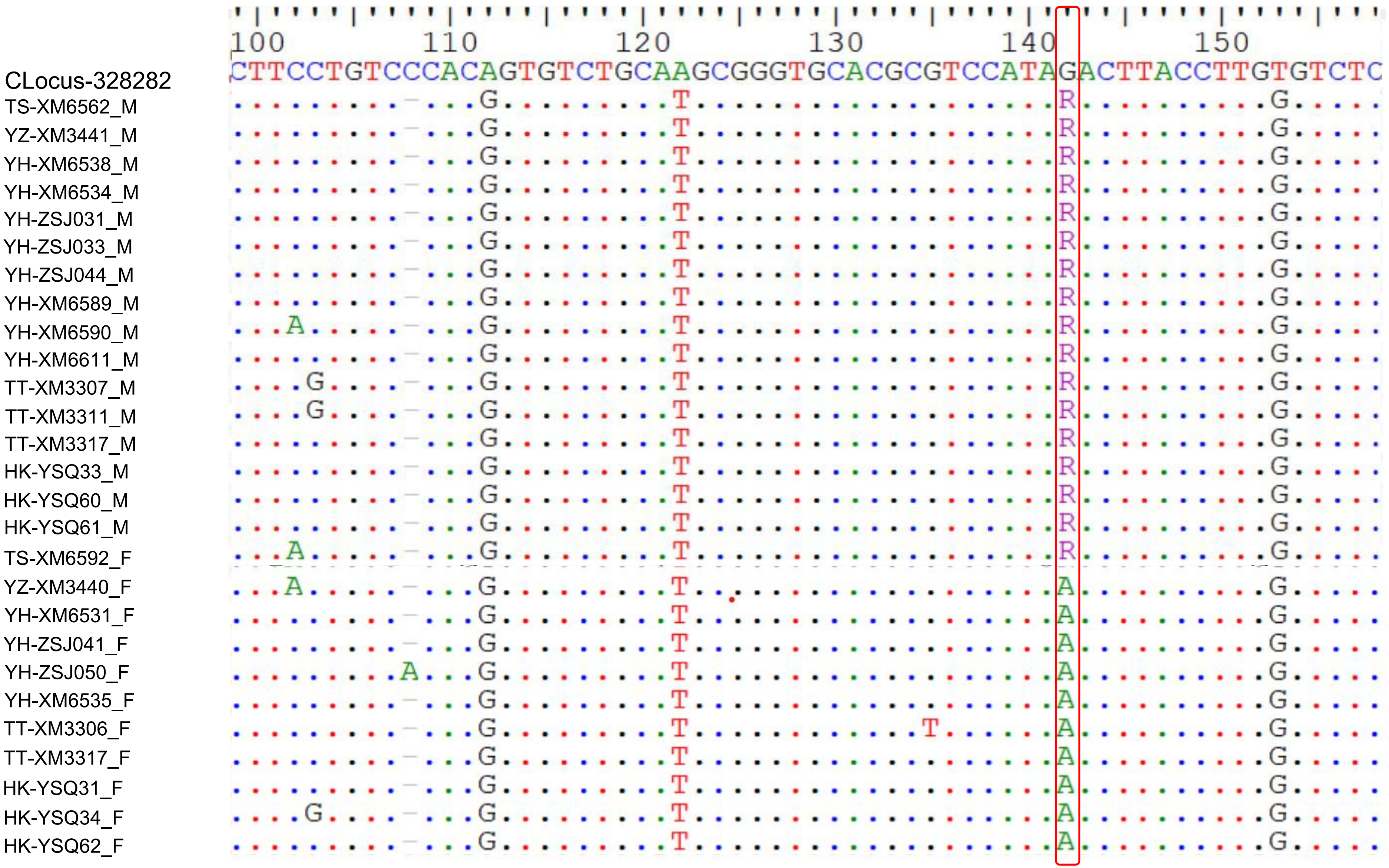

100
110
120
130
140
150
CLocus-328282
CTTCCTGTCCCACAGTGTCTGCAAGCGGGTGCACGCGTCCATAGACTTACCTTGTGTCTC
TS-XM6562_M
YZ-XM3441_M
YH-XM6538_M
YH-XM6534_M
YH-ZSJ031_M
YH-ZSJ033_M
YH-ZSJ044_M
YH-XM6589_M
YH-XM6590_M
YH-XM6611_M
TT-XM3307_M
TT-XM3311_M
TT-XM3317_M
HK-YSQ33_M
HK-YSQ60_M
HK-YSQ61_M
TS-XM6592_F
YZ-XM3440_F
YH-XM6531_F
YH-ZSJ041_F
YH-ZSJ050_F
YH-XM6535_F
TT-XM3306_F
TT-XM3317_F
HK-YSQ31_F
HK-YSQ34_F
HK-YSQ62_F

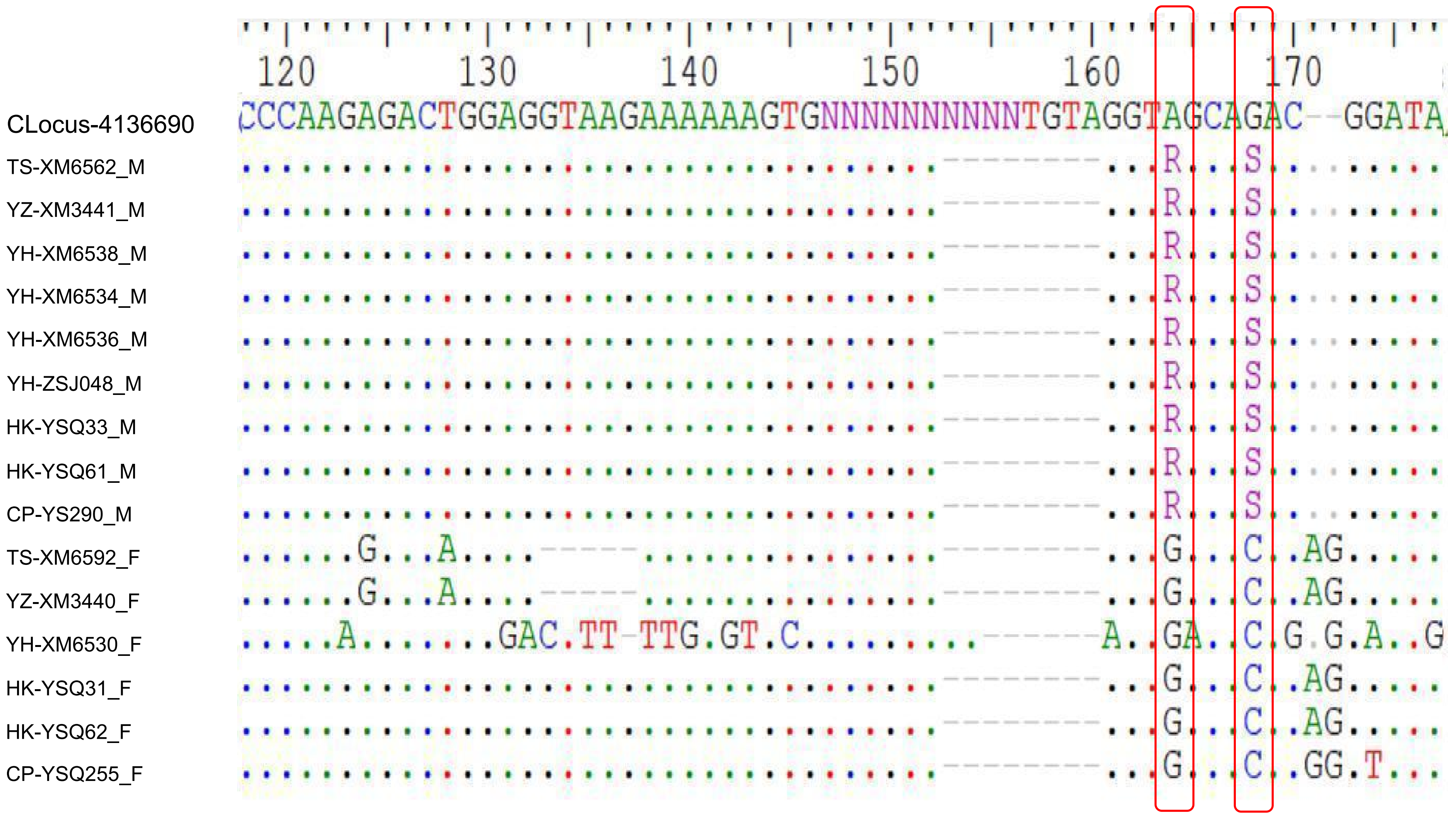
120
130
140
150
160
170
CLocus-4136690
TS-XM6562_M
YZ-XM3441_M
YH-XM6538_M
YH-XM6534_M
YH-XM6536_M
YH-ZSJ048_M
HK-YSQ33_M
HK-YSQ61_M
CP-YS290_M
TS-XM6592_F
YZ-XM3440_F
YH-XM6530_F
HK-YSQ31_F
HK-YSQ62_F
CP-YSQ255_F

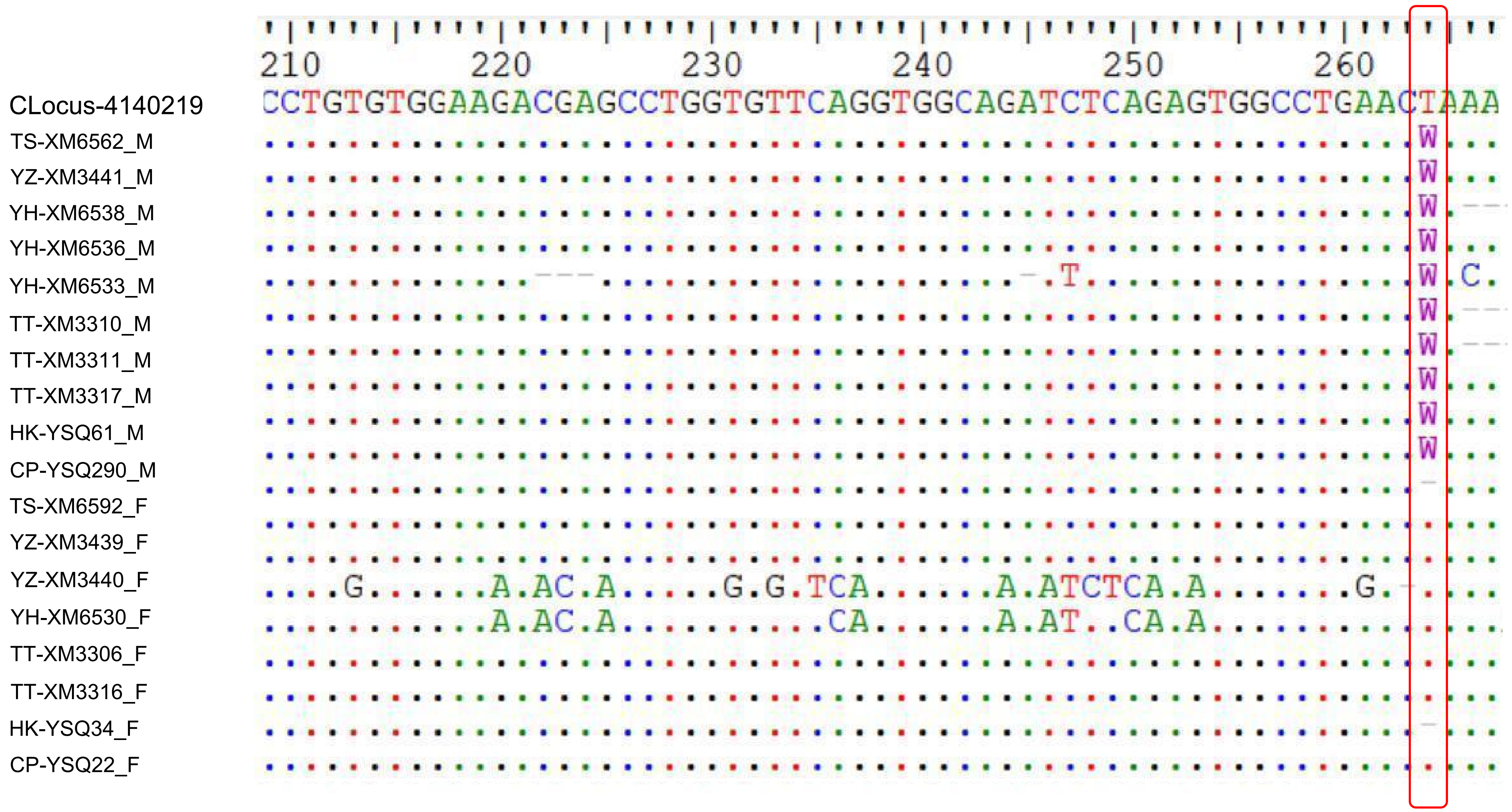
210
220
230
240
250
260
CLocus-4140219
CCTGTGTGGAAGACGAGCCTGGTGTTCAGGTGGCAGATCTCAGAGTGGCCTGAACTAAA
TS-XM6562_M
YZ-XM3441_M
YH-XM6538_M
YH-XM6536_M
YH-XM6533_M
TT-XM3310_M
TT-XM3311_M
TT-XM3317_M
HK-YSQ61_M
CP-YSQ290_M
TS-XM6592_F
YZ-XM3439_F
YZ-XM3440_F
YH-XM6530_F
TT-XM3306_F
TT-XM3316_F
HK-YSQ34_F
CP-YSQ22_F

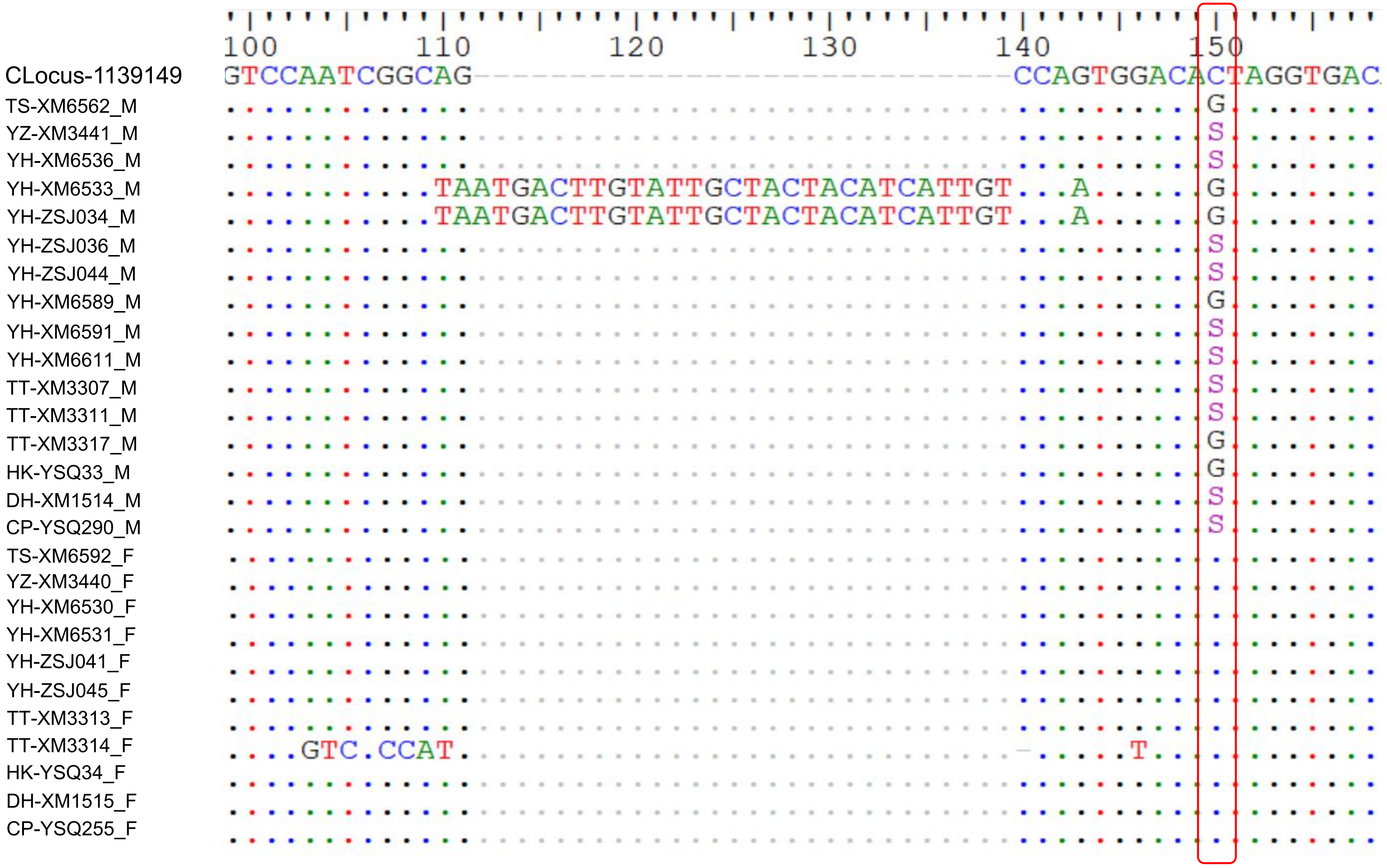
100
110
120
130
140
150
CLocus-1139149
GTCCAATCGGCAG
CCAGTGGACACTAGGTGAC
TS-XM6562_M
YZ-XM3441_M
YH-XM6536_M
YH-XM6533_M
YH-ZSJ034_M
YH-ZSJ036_M
YH-ZSJ044_M
YH-XM6589_M
YH-XM6591_M
YH-XM6611_M
TT-XM3307_M
TT-XM3311_M
TT-XM3317_M
HK-YSQ33_M
DH-XM1514_M
CP-YSQ290_M
TS-XM6592_F
YZ-XM3440_F
YH-XM6530_F
YH-XM6531_F
YH-ZSJ041_F
YH-ZSJ045_F
TT-XM3313_F
TT-XM3314_F
HK-YSQ34_F
DH-XM1515_F
CP-YSQ255_F
TAATGACTTGTATTGCTACTACATCATTGT
GTC
CCAT

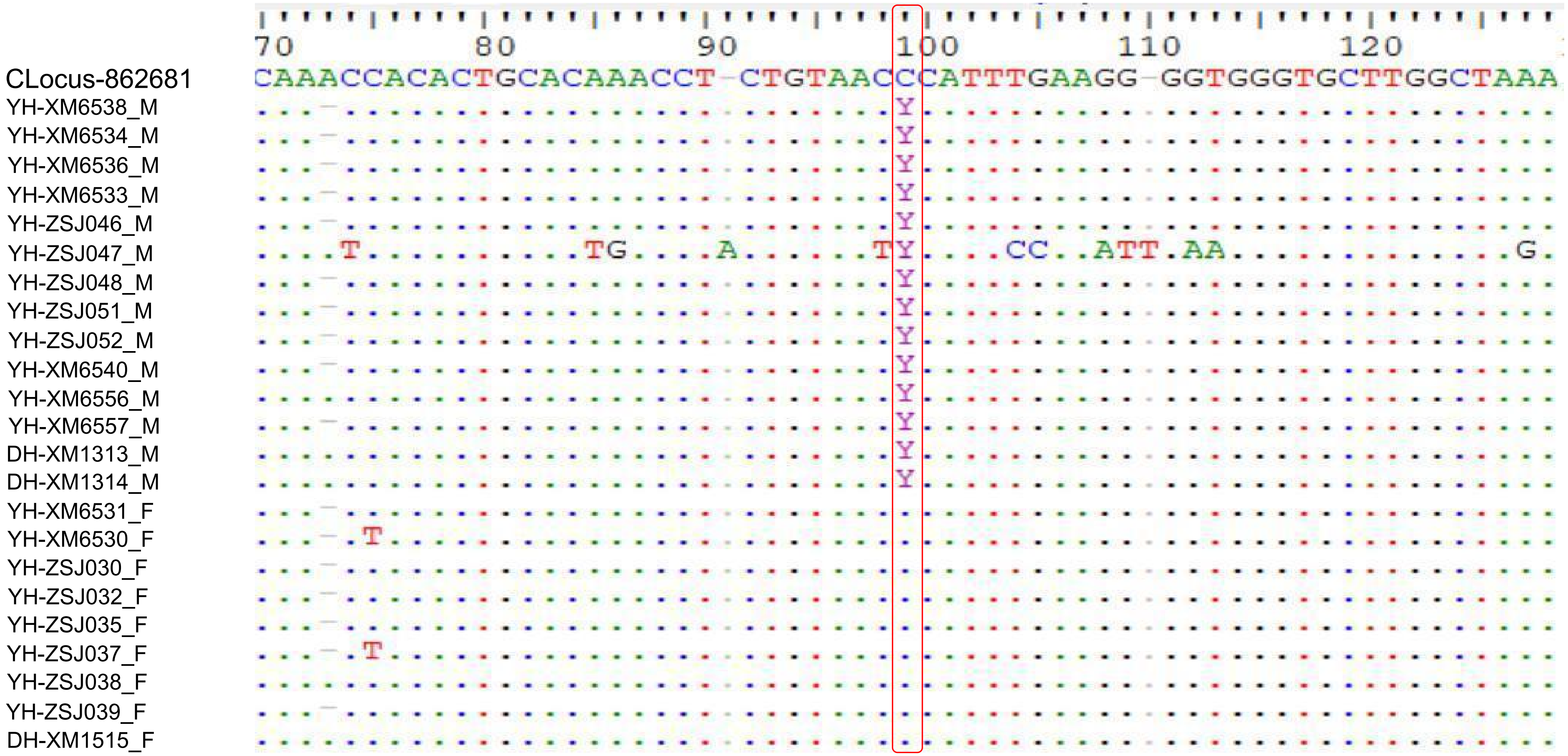
70
80
90
100
110
120
CLocus-862681
CAAACCACACTGCACAAACCT-CTGTAACCCATTTGAAGG-GGTGGGTGCTTGGCTAAA
YH-XM6538_M
YH-XM6534_M
YH-XM6536_M
YH-XM6533_M
YH-ZSJ046_M
YH-ZSJ047_M
YH-ZSJ048_M
YH-ZSJ051_M
YH-ZSJ052_M
YH-XM6540_M
YH-XM6556_M
YH-XM6557_M
DH-XM1313_M
DH-XM1314_M
YH-XM6531_F
YH-XM6530_F
YH-ZSJ030_F
YH-ZSJ032_F
YH-ZSJ035_F
YH-ZSJ037_F
YH-ZSJ038_F
YH-ZSJ039_F
DH-XM1515_F
