# Supplementary material for: Identification of Male-Specific Markers by Genotyping-by-Sequencing in the Giant Spiny Frog, Quasipaa spinosa
Source: Genes (Basel). 2025 Nov 7;16(11):1347. doi: 10.3390/genes16111347 (PMC12652585; doi:10.3390/genes16111347)
Supplement: Supplementary file 1 [file genes-16-01347-s001.zip › Figure S3..pdf]

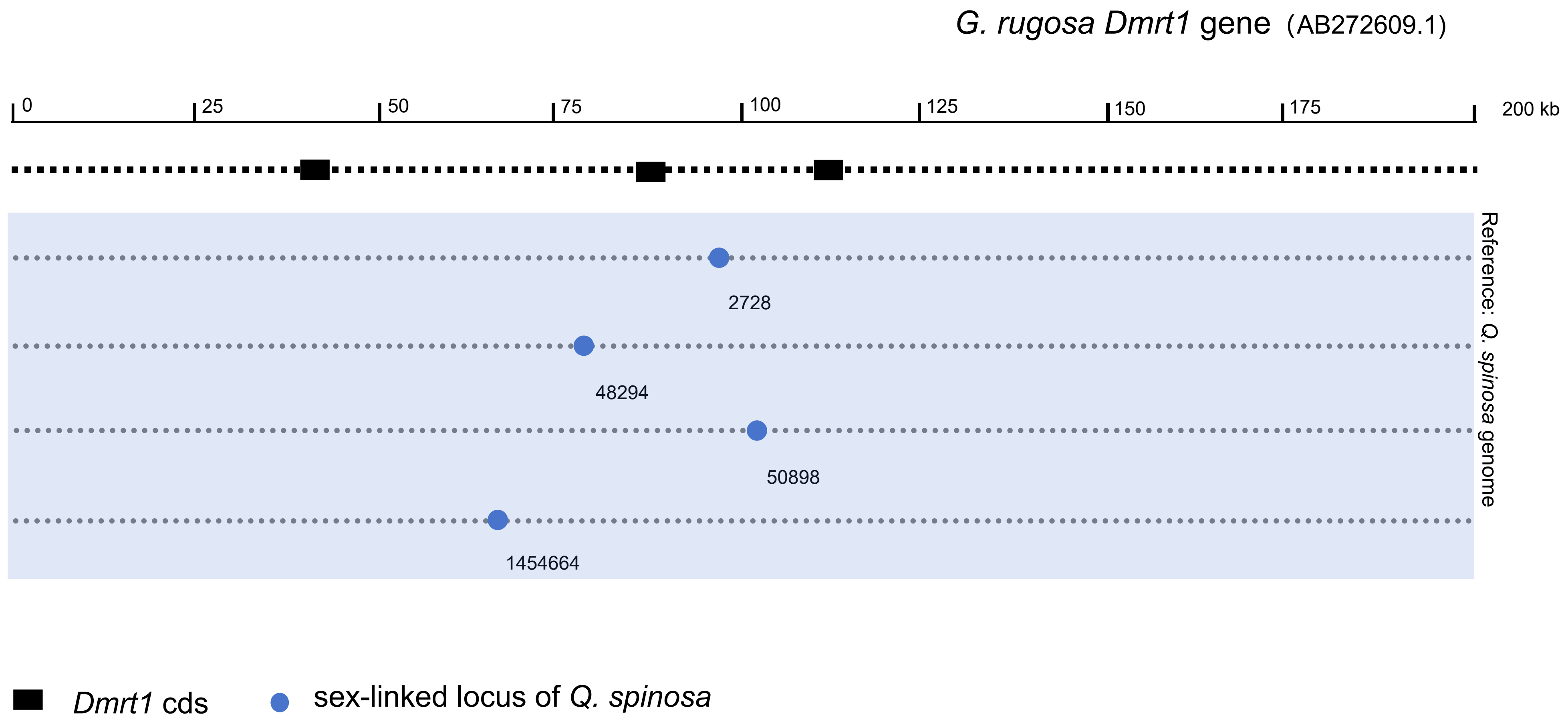

Figure S3. The mapping of sex-linked locus on *DMRT1* gene of *G. rugosa*. Circles indicate sex-linked loci of *Q. spinosa*, dashed lines indicate the relative alignment region of the *DMRT1* gene of *G. ruguosa* in the *Q. spinosa* genome.
