# Supplementary material for: Identification of Male-Specific Markers by Genotyping-by-Sequencing in the Giant Spiny Frog, Quasipaa spinosa
Source: Genes (Basel). 2025 Nov 7;16(11):1347. doi: 10.3390/genes16111347 (PMC12652585; doi:10.3390/genes16111347)
Supplement: Supplementary file 1 [file genes-16-01347-s001.zip › File S1.pdf]

File S1. Sample size and location information.

Male individuals are represented by the symbol “M” and female individuals by the symbol “F”. GBS sequencing was performed on the underlined individuals. Each geographic population is assigned a short code for easier reference in the text (e.g., TS stands for Tongshan County). The voucher specimens were deposited in the Herpetological Museum of Chengdu Institute of Biology, Chinese Academy of Sciences.

1. Tongshan County, Xianning City, Hubei Province, China. (TS, n= 2, 1F, 1M. N 29.436886°, E 114.677381°)

XM6562M, XM6592F.

2. Yizhang County, Chenzhou City, Hunan Province, China. (YZ, n=3, 2F, 1M. N 24.957500° , E 12.975833°)

XM3439F, XM3440F, XM3441M.

3. Yihuang County, Fuzhou City, Jiangxi Province, China. (YH, n=37, 22M, 17F, N 27.302222° , E 116.125036°)

ZSJ030F, ZSJ031M, ZSJ032F, ZSJ033M, ZSJ034M, ZSJ035F, ZSJ036M, ZSJ037F, ZSJ038F, ZSJ039F, ZSJ040F, ZSJ041F, ZSJ042F, ZSJ043M, ZSJ044M, ZSJ045F, ZSJ046M, ZSJ047M, ZSJ048M, ZSJ049F, ZSJ050F, ZSJ051M, ZSJ052M, XM6530F, XM6531F, XM6533F, XM6534M, XM6535F, XM6536M, XM6537F, XM6538M, XM6540M, XM6556M, XM6557M, XM6589M, XM6590M, XM6591M, XM6610M, XM6611M.

4. Tiantai County, Taizhou City, Zhejiang Province, China. (TT, n=10, 5F, 5M, N 25.654823° , E 118.217188°)

XM3306F, XM3307M, XM3310M, XM3311M, XM3312F, XM3313F, XM3314F XM3315M, XM3316F, XM3317M.

5. Huangkeng Town, Jianyang District, Nanping City, Fujian Province, China. (HK, n=6, 4M, 2F, N 27.692361° , E 117.649825°)

YSQ31F, YSQ33M, YSQ34F, YSQ60M, YSQ61M, YSQ62M.

6. Dehua County, Quanzhou City, Fujian Province, China. (DH, n=3, 2M, 1F, N 25.664967° , E 118.219350°)

XM1513M, XM1514M, XM1515F.

7. Ciping Town, Suichuan County, Jinggangshan City, Jiangxi Province, China. (CP, n=4, 3M, 1F, N 26.5110278°, E 114.0977139°)

YSQ290M, YSQ253M, YSQ254M, YSQ255F.
